# Associations between cumulative risk, childhood sleep duration, and body mass index across childhood

**DOI:** 10.1186/s12887-022-03587-6

**Published:** 2022-09-06

**Authors:** Tiffany Phu, Jenalee R. Doom

**Affiliations:** Department of Psychology, University of Denver, 2155 South Race St, Denver, CO 80210 USA

**Keywords:** Body mass index, Child health, Overweight, Sleep, Cumulative risk

## Abstract

**Background:**

Although associations between cumulative risk, sleep, and overweight/obesity have been demonstrated, few studies have examined relationships between these constructs longitudinally across childhood. This study investigated how cumulative risk and sleep duration are related to current and later child overweight/obesity in families across the United States sampled for high sociodemographic risk.

**Methods:**

We conducted secondary analyses on 3690 families with recorded child height and weight within the Fragile Families and Child Well-Being Study. A cumulative risk composite (using nine variables indicating household/environmental, family, and sociodemographic risk) was calculated for each participant from ages 3-9 years. Path analyses were used to investigate associations between cumulative risk, parent-reported child sleep duration, and z-scored child body mass index (BMI) percentile at ages 3 through 9.

**Results:**

Higher cumulative risk experienced at age 5 was associated with shorter sleep duration at year 9, b = − 0.35, *p* = .01, 95% CI [− 0.57, − 0.11]. At 5 years, longer sleep duration was associated with lower BMI, b = − 0.03, *p* = .03, 95% CI [− 0.06, − 0.01]. Higher cumulative risk at 9 years, b = − 0.34, *p* = .02, 95% CI [− 0.57, − 0.10], was concurrently associated with shorter sleep duration. Findings additionally differed by child sex, such that only male children showed an association between sleep duration and BMI.

**Conclusions:**

Results partially supported hypothesized associations between child sleep duration, cumulative risk, and BMI emerging across childhood within a large, primarily low socioeconomic status sample. Findings suggest that reducing cumulative risk for families experiencing low income may support longer child sleep duration. Additionally, child sleep duration and BMI are concurrently related in early childhood for male children.

**Supplementary Information:**

The online version contains supplementary material available at 10.1186/s12887-022-03587-6.

About a third of children in the United States have overweight or obesity [1]. Childhood overweight/obesity predicts adult overweight/obesity and is associated with adult morbidity (e.g., higher rates of diabetes, coronary heart disease, and some types of cancer) [2]. Therefore, effectively utilizing levers of change to reduce childhood overweight/obesity may critically change trajectories of lifelong physical health. Drawing from Bronfenbrenner’s bioecological theory [3], children are situated within contexts (e.g., families, neighborhoods) that influence development. This attention to how ecological systems intersect with child developmental processes has been extended to overweight/obesity, in recognition of how family and community characteristics may shape child biological regulation and behavioral patterns that transmit overweight/obesity [4]. The current study seeks to examine pediatric weight in context of both ecological systems and child sleep, an important biobehavioral process linked to satiety and hormonal systems relevant to weight [5].

## Overweight/obesity and cumulative risk

Overweight/obesity is more prevalent in contexts of high stress, such as poverty: about 15% of children ages 2-4 years from low-income backgrounds meet criteria for obesity [6]. Children in families and neighborhoods experiencing low-income demonstrate greater likelihood of overweight/obesity [7], potentially due to increased exposure to psychosocial stressors [8, 9], or to physical characteristics of the built environment, such as high residential density and low available supermarkets [10]. Relevant to the current study, higher cumulative risk in middle childhood has been linked to increased adolescent BMI among a rural sample [11]; we extend this literature by studying a primarily urban, younger sample of children and examining sleep.

### Cumulative risk

Evans proposed cumulative risk [12] as a measurement technique to capture multiple risk exposures, including sociodemographic (e.g., poverty, single parent), psychosocial (e.g., familial contextual factors such as parental mental health, exposure to violence), and environmental (e.g., physical conditions such as high noise, household crowding) risk factors [11]. Cumulative risk places a greater premium on high levels of risk by only assigning a dichotomous indicator of risk exposure to the upper end of exposure. In this way, cumulative risk intends to capture the experience of elevated stress.

The current literature indicates a range of negative health and psychosocial outcomes that are linked to experiencing higher cumulative risk. Higher cumulative risk has been longitudinally associated with more negative psychological, educational, and health-related outcomes (e.g., depression, school dropout, physical disease) in young adulthood [13]. Within childhood, higher cumulative risk is associated with concurrently poor sleep health and sleep disorder symptoms [14]. Higher cumulative risk in childhood also predicts increased behavior problems in adolescence [15]. Some evidence indicates that cumulative risk may mediate the effects of stressors on physiological functioning. For example, higher cumulative risk has been found to transmit the effects of poverty exposure on increased physiological reactivity to stressors [16] and brain activation in emotion regulation areas [17]. Taken together, cumulative risk may negatively impact weight by conferring increased physiological dysregulation, difficulties in emotion regulation, and exposure to stressors. Indeed, cumulative risk in middle childhood is associated with diminished self-regulatory capacity and subsequently higher adolescent BMI [18].

## Sleep intersects with cumulative risk and overweight/obesity

Sleep is regulated by both biology and behavior. Important dimensions of pediatric sleep health are posited as duration, satisfaction, alertness, timing, efficiency, and sleep-related behaviors [19]. Sleep health has been linked to physiologic benefits, particularly as reflected by healthier stress response pattern [20]. Sleep may influence risk for obesity via both physiological and behavioral pathways. For example, greater sleep disturbances decrease circulating levels of leptin, a hormone that regulates fat storage and inhibits hunger, disrupting appetite regulation [21]. Preschool children with poor sleep health demonstrate higher caloric intake of sugar and carbohydrates [22]. Short sleep duration in early childhood has been prospectively linked to increased risk of later overweight/obesity [23]. In the current study, we focus on sleep duration as one indicator of pediatric sleep health.

Sleep problems and poor sleep health are more likely to occur in contexts of both pediatric overweight/obesity and high cumulative risk [24]. Children in families experiencing higher levels of cumulative risk demonstrate more sleep problems compared to those in families with low risk levels [25]. Even within families experiencing low-income, greater cumulative risk is positively associated with sleep problems for toddlers [26]. Poor child sleep health may increase risk for later overweight/obesity following early exposure to cumulative risk [23]. Taken together, biological and behavioral pathways link sleep and overweight/obesity in children (for an overview of biological linkages, see [27]), though investigations of longitudinal associations between all these factors are needed.

### Prior work in the fragile families and child wellbeing study (FFCWS)

Suglia and colleagues [28] found that cumulative social risk at age 3, characterized by maternal report of intimate partner violence, food insecurity, housing insecurity, maternal depressive symptoms, maternal substance use, and paternal incarceration, was associated with obesity for girls at age 5. After adjusting for behavioral problems and sociodemographic factors (i.e., maternal race/ethnicity, maternal education, parental marital status, and receipt of public assistance), a concurrent association between short sleep duration and increased odds of obesity at age 5 emerged. Duarte et al. [29] analyzed environmental conditions of interior and exterior household characteristics (e.g., broken windows, cluttered rooms, peeling paint, crumbling walls), finding a cross-sectional association with poorer environmental conditions and greater child z-scored BMI at age 3. The current investigation using FFCWS data will expand on these studies to include a broader measurement of cumulative risk (i.e., household and environmental risk, family risk, and sociodemographic risk) and repeated assessments of sleep and BMI across three time points. Operationalizing cumulative risk across psychosocial factors and environmental conditions may allow increased accuracy in capturing factors that are known to potentiate physiological and psychosocial stress.

### Race/ethnicity as an important context

Nationally representative studies in the United States indicate racial/ethnic disparities in pediatric overweight/obesity rates, such that Black, Latinx, and Native American children demonstrate higher overweight/obesity rates compared to their White and Asian counterparts [30]. Race/ethnicity can denote different social, cultural, and/or environmental contexts that shape overweight/obesity [31]. Health researchers have broadly pointed to structural racism as a fundamental cause of racial health inequities, perpetuated by factors such as discriminatory housing and carceral policies [32], that have thus imbued racial/ethnic groups as a meaningful social construct to examine when considering health.

## Current study and rationale

The present study examines longitudinal associations of cumulative risk and sleep on child BMI in urban children and families across the United States participating in the FFCWS. We extend prior work by testing transactional and cascading effects of cumulative risk and sleep on BMI at ages 3, 5, and 9 years to understand how these developmental processes unfold in children experiencing stressors. We use autoregressive and lagged paths in a path analysis to model cumulative risk, sleep, and BMI at each timepoint, which allows for examining associations between variables over time while controlling for effects at earlier timepoints. Multi-group analyses were also run to examine the analytic model by maternal race/ethnicity and acknowledge the rich racial/ethnic diversity of the FFCWS sample and race/ethnicity as an important socioecological context in the United States. As prior studies have indicated sex differences in relationships between risk, sleep, and obesity [28, 33], analyses were also conducted by child sex to test different pathways from cumulative risk, shorter sleep duration, and BMI. One specific hypothesis was that a cascading pathway would emerge between cumulative risk at age 3 to sleep at year 5 and BMI at year 9.

Examining how cumulative risk and sleep duration may shape BMI can inform potential intervention targets for families experiencing high sociodemographic stressors, who demonstrate higher rates of pediatric overweight/obesity [7]. BMI is an important aspect of child physical health that has been linked to adult morbidity and premature mortality [34]. Therefore, investigating potential supports for healthy childhood BMI (e.g., sleep health, reducing cumulative risk) within a sample experiencing high sociodemographic risk is warranted.

## Method

### Fragile families and child well-being study

Data derive from FFCWS, a prospective birth cohort study of 4898 urban parents and their infants born between 1998 and 2000 across 20 sites in the United States [35]. Unmarried parents were oversampled, representing about three-quarters of the sample. Biological parents (mothers and fathers) were interviewed about socioeconomic status, health, employment, social support, parenting, and relationship status shortly after the child’s birth. Information on child health and well-being were collected at ages 0, 3, 5, and 9 years. Child sleep duration and BMI were measured within the same study visit. Detailed information about FFCWS’s sampling design and methodology are described in Reichman et al. [35]. All participants provided informed consent. FFCWS procedures were approved by affiliated human subjects review board at data collection sites. The current secondary data analysis was reviewed by the University of Denver’s Institutional Review Board and determined to not meet the federally regulated definition of human subjects research; therefore, ethics approval was waived.

The present study utilizes data from birth through 9 years. The analytic sample included 3690 families with measured child height and weight across at least one of the three timepoints and reported information on maternal race/ethnicity and child low birthweight (See Table 1 for demographics). Additionally, only participants with at least five or more indicators reported out of a possible nine used for the cumulative risk composite were included (85% of the sample at age 3, 83% at age 5, and 72% at age 9). This is a helpful but limited approach in managing missing data, as attrition in follow-up studies may be related to socioeconomic inequalities [36]. When comparing demographics of those with at least five or more risk indicators versus those who did not within the full FFCWS sample, participants with sufficient cumulative risk data did not differ on child sex χ^2^(1) = .38, *p* = .54, had slightly higher income-to-needs ratio *t*(4895) = 1.60, *p* = .06 (those ≥5 indicators, *M* = 2.24, *SD =* 2.42; < 5 indicators, *M* = 2.01, *SD* = 2.27), were more likely to have a high school education, χ^2^(1) = 10.67, *p* = .001 (≥ 5 indicators, 66% high school education and above; < 5 indicators, 57% high school education and above), and differential distribution of maternal race/ethnicity, χ^2^(3) = 15.96, *p* = .001, (≥ 5 indicators, 48% non-Hispanic Black, 27% Latinx, 21% non-Hispanic White, 4% Other; < 5 indicators, 41% non-Hispanic Black, 32% Latina, 19% non-Hispanic White, 8% Other).

**Table 1:** Demographic and descriptive statistics

	M (SD) or Percentage (%)		
	Wave 3 (age 3)	Wave 4 (age 5)	Wave 5 (age 9)
**Demographics**	*n* = 3451	*n* = 3419	*n* = 3229
Child age (years)	2.97 (0.21)	5.14 (0.23)	9.38 (0.37)
Child sex (female)	48.3%	48.0%	48.0%
Maternal age (years)	27.97 (5.97)	30.09 (5.96)	34.30 (5.95)
Maternal race/ethnicity			
White	20.8%	20.2%	20.9%
Black	50.1%	50.7%	50.2%
Latina	25.9%	25.9%	25.7%
Other	3.2%	3.1%	3.2%
**Study Constructs**			
Child BMI Percentile	63.73 (30.61)	66.36 (28.56)	68.14 (27.93)
Categorical BMI			
Underweight	4.4%	3.1%	2.2%
Average	60.8%	62.1%	56.3%
Overweight	16.9%	17.5%	16.8%
Obese	17.9%	17.3%	24.8%
Child Sleep Duration (hours)	–	9.40 (1.28)	8.95 (1.12)
Cumulative Risk	0.23 (0.19)	0.21 (0.18)	0.20 (0.17)

### Measures

#### Cumulative risk

Cumulative risk composites were created for ages 3, 5, and 9 years following the conceptual framework of Evans et al. [37]. Nine risk variables were dichotomously coded at each wave and summed across household/environmental, family, and sociodemographic domains. Using a cumulative risk measurement approach may be beneficial due to its parsimony, reduced measurement error, and insensitivity to risk collinearity [37]. A wealth of prior studies have used this cumulative risk measurement approach, finding relationships to pediatric sleep health indices [14], pediatric obesity [18], and child well-being [13]. Risk variables were derived from trained research staff observations or by primary caregiver report. To account for missing data, we divided the number of endorsed risk indicators over the total possible number of items. Therefore, the cumulative risk composite had a possible range of 0-1, with higher values indicating greater endorsed risk. Continuous variables (i.e., substandard housing, crowding, family turmoil) were dichotomously coded, with 1 representing a value greater than 1 SD above the mean for that risk factor type. Descriptive statistics are provided in Table 2. Individual variables for the cumulative risk measure are described below.

**Table 2:** Descriptive statistics for cumulative risk variables

	M (SD) or %		
	Age 3 years	Age 5 years	Age 9 years
**Household or environmental risk variables**			
Substandard housing conditions and hazards sum	1.05 (1.96)	1.03 (1.89)	0.76 (1.63)
Exterior conditions	0.94 (1.67)	0.92 (1.65)	0.70 (1.39)
Interior conditions	0.27 (0.68)	0.21 (0.57)	0.11 (0.41)
Interior hazards	0.06 (0.43)	0.07 (0.38)	1.48 (1.77)
Household size (Total household members)	4.36 (1.62)	4.44 (1.61)	4.63 (1.62)
Noise (Endorsed “Very Noisy”)	15.8%	22.3%	24.0%
**Family risk variables**			
Child separation from biological parents (yes)	11.2%	10.2%	11.4%
Child exposure to violence (yes)	13.5%	10.6%	8.2%
Family turmoil	0.67 (0.94)	0.65 (0.96)	0.64 (0.91)
Housing insecurity	20.1%	20.4%	25.4%
Food insecurity	14.0%	7.1%	7.2%
Maternal alcohol/drug use	10.2%	9.1%	30.9%
Maternal depression	20.6%	17.0%	17.5%
Paternal incarceration	8.1%	8.3%	6.5%
Maternal intimate partner violence	18.4%	6.3%	5.4%
**Sociodemographic risk variables**			
Maternal education- less than high school	27.9%	25.9%	22.0%
Single mother	46.7%	51.8%	52.4%
Socioeconomic disadvantage (Income-to-needs ratio)	1.94 (2.53)	1.92 (2.25)	1.99 (2.29)

### Household or environmental risk variables

#### Substandard housing conditions and hazards

Trained observers dichotomously rated the household physical conditions for housing conditions and hazards, with nine exterior conditions (e.g., broken features, strewn garbage/litter, peeling paint), four interior conditions (e.g., cracks, exposed wires), and six interior hazards (e.g., vermin, exposed plaster). These 19 dichotomous items were summed to create a composite indicating substandard housing conditions and hazards. This composite was then dichotomously coded, with 1 representing a value greater than 1 SD above the mean. Housing items were selected for use in FFCWS due to prior work with similar items indicating links to psychological distress [38].

#### Household size

Household size was measured as the total reported number of adults and children in the household, as a proxy for household crowding. Parents were not asked to report total number of bedrooms at age 9, and thus total household members is used as a proxy for crowding to use multiple measurements across waves. A systematic review found that people per household was the second most utilized measure of household crowding [39]; it is also positively correlated with people per bedroom [40]. Household size was coded as 1 if the total number of household members was 1 SD above the mean.

#### Noise

Parents were asked one question to assess noise: “how noisy is the house or apartment”. Response options included “not very noisy,” “somewhat noisy,” or “very noisy”. Noise was coded as 1 if parents described the house or apartment as “very noisy”.

### Family risk variables

#### Child separation from parents

Parents reported how much of the time child lived with them. If the child lived with parents “None of the time”, “Sometimes”, or “Half time” (compared to “Most of the time”) and parents reported that a grandparent, foster parent, or “other” person usually lives with child (compared to the other biological parent), child separation from parents was coded as 1.

#### Child exposure to violence

Child exposure to violence was coded as 1 if parents reported physically fighting with one another with the child present.

#### Family turmoil

Six dichotomous or dichotomously-coded items (i.e., housing insecurity, food insecurity, maternal drug/alcohol use, probable maternal depression, and paternal incarceration) were summed to create a family turmoil risk factor, following prior work operationalizing a cumulative social risk index in FFCWS [28]. This family turmoil composite sum was coded as 1 if more than 1 SD above the sample mean. Housing and food insecurity questions derived from the New York City Social Indicators Survey [41]. Mothers were dichotomously characterized as experiencing housing insecurity if, in the past year, they endorsed any of the following: (a) eviction, (b) staying in a shelter/car, (c) not paying full rent/mortgage, or (d) moving in with others due to financial problems. Mothers were dichotomously characterized as experiencing food insecurity if, in the past year, they endorsed either (a) inability to afford more food despite hunger or (b) their children being hungry. Maternal substance use and depression were assessed with questions based on the Composite International Diagnostic Interview- Short Form (CIDI-SF; [42]). Internal consistency of an adapted CIDI-SF questionnaire has been estimated as α = .67 for substance use and α = .87 for depression [43]. Mothers were classified as having problematic use of drugs/alcohol if they endorsed any of the following in the past year: (a) drinking more than 4-5 alcoholic beverages in 1 day, (b) smoking pot/marijuana, (c) using hard drugs, (d) seeking help or treatment due to drug/alcohol problems, or that (e) drugs/alcohol interfered with their daily life or personal relations. Probable depression within the past year was coded as 1 if mothers reported experiencing daily dysphoria or anhedonia across at least 2 weeks and reported three additional depressive symptoms (out of a possible seven). Paternal incarceration was coded as 1 if parents reported that the biological father was currently incarcerated. Maternal intimate partner violence was coded as 1 if mother reported that the child’s father or mother’s romantic partner “Often” or “Sometimes” (versus “Never”) slapped or kicked her, hit her with a fist or dangerous object, or forced sex on her within the past month.

### Sociodemographic risk variables

#### Maternal education— less than high school

Mothers reported highest level of education attained, which was coded as a 1 if it was less than high school.

#### Single mother

Mothers responded to questions on their current relationship status with the baby’s father or another romantic partner. Mothers were characterized as single parents and coded as a 1 if they indicated that they were not married or cohabitating with the child’s father or another romantic partner. Prior research suggests that cohabitation may more accurately represent parental involvement in childrearing and shared allocation of household resources than marital status [44].

#### Socioeconomic disadvantage

Income-to-needs ratio was calculated using mother-reported household income divided by the federal poverty line for a family of that size [45]. An income-to-needs ratio that was less than or equal to one was coded as 1 for experiencing socioeconomic disadvantage.

##### Child sleep

Mothers reported on child sleep duration at child ages 5 and 9 by answering, “How many hours of sleep a night does your child usually get during the week?”. Mother-reported child sleep duration has shown adequate validity and test-retest reliability [46]. We elected to focus on sleep duration as an indicator for overall sleep health due to solid evidence that duration is associated with a wide range of health indices in early childhood [47].

##### Child BMI

Child height and weight were measured by trained research assistants at ages 3, 5, and 9 years using a portable stadiometer for height and an electronic scale for weight. Children wore light clothing and no shoes. BMI was calculated using child height and weight (kg/m^2^). The Center for Disease Control and Prevention (CDC) growth reference charts were applied to determine age- and gender-specific BMI percentiles [48], then z-scored for analysis. BMI percentile was categorized for descriptive purposes, consistent with CDC guidelines: Underweight ≤5th percentile, Average = 6th to 84th percentile; Overweight = 85th to 94th percentile; Obese ≥95th percentile.

##### Covariates

Mothers reported on child sex and maternal race (i.e., Black, White, Asian, American Indian, Other non-identified) and ethnicity (i.e., Hispanic, non-Hispanic) shortly after child’s birth. Maternal race/ethnicity was categorized into: non-Hispanic Black, Latina, non-Hispanic White, or Other (i.e., Asian, American Indian, Other non-identified). Mothers also reported on child birthweight shortly after child’s birth. Consistent with World Health Organization guidelines [49], birthweight was dichotomously categorized low (1) if birthweight was less than 2500 g or healthy (0) if above. In this sample, 9.9% of children were identified as low birthweight.

### Analytic plan

Path analyses were run in M*plus*, version 8.4 [50] to test relations between cumulative risk, sleep, and BMI percentile using timepoints from ages 3 through 9. Potential covariates (i.e., child sex, child age, child low birthweight, dummy coded maternal race/ethnicity) were assessed using a correlation matrix (see Table 3). Covariates associated with main study variables were entered in the model on sleep, BMI, and cumulative risk at each timepoint. The final model included paths between the same constructs measured at different timepoints (i.e., autoregressive paths) and paths between prior time-point variation across sleep, BMI, and cumulative risk (i.e., lagged paths). All lagged paths except for prior sleep and BMI on subsequent cumulative risk were entered.

**Table 3:** Pearson’s correlation coefficients between variables of interest

	1	2	3	4	5	6	7	8	9	10	11	12	13	14	15	16	17
1. BMI y3	1																
2. BMI y5	.55^***^	1															
3. BMI y9	.47^***^	.66^**^	1														
4. Sleep duration y5	−.05^*^	−.03	−.03^+^	1													
5. Sleep duration y9	−.01	.02	−.01	.39^***^	1												
6. Risk y3	.01	.02	.03	−.02	−.04^**^	1											
7. Risk y5	−.01	.02	.04^*^	.01	−.05^**^	.64^***^	1										
8. Risk y9	.00	.03	.03	−.07^***^	−.16^***^	.54^***^	.58^***^	1									
9. Child age y3	.05^*^	.03	.04^*^	−.24^**^	−.11^***^	.13^**^	.13^**^	.12^**^	1								
10. Child age y5	.05^*^	.02	.03	−.32^***^	−.14^***^	.07^***^	.08^***^	.06^***^	.36^***^	1							
11. Child age y9	.03	.04^+^	.02	−.30^***^	−.08^***^	.10^***^	.12^***^	.13^***^	.29^***^	.29^***^	1						
12. Child sex- Female	.01	.01	.00	.01	.00	0.01	−.01	.02	.00	.02	.02	1					
13. Low birth weight (Yes/no)	−.01	.01	.01	.01	.00	−.01	.02	.00	.01	.00	−.04^*^	.01	1				
14. White	−.01	−.04^+^	−.12^***^	.02	.04^*^	−.25^***^	−.25^***^	−.24^***^	−.16^***^	−.13^***^	−.09^***^	.00	−.01	1			
15. Black	−.10^***^	−.06^*^	.04^*^	.07^***^	.05^**^	.16^***^	.17^***^	.17^***^	.08^***^	.03^*^	−.05^**^	.00	.02	−.49^***^	1		
16. Latinx	.13^***^	.11^***^	.07^***^	−.05^***^	.07^***^	.08^***^	.07^***^	.05^**^	.05^***^	.09^***^	.12^***^	.01	.00	.31^***^	−.59^***^	1	
17. Other	−.02	−.03	−.02	−.07^***^	−.04^**^	−.06^***^	−.07^***^	−.06^***^	−.01	.00	.03^+^	.00	.00	−.11^***^	−.19^***^	−.13^***^	1

*Note*: * = *p* < .05, ** = *p* < .01, *** = *p* < .001, + = *p* < .10. *BMI* Body mass index percentile. *Risk* cumulative risk. Race/ethnicity was of mother

Group differences in the main constructs of interest (cumulative risk, sleep, BMI) by maternal race/ethnicity were tested. Maternal race/ethnicity, child sex, and categorical BMI percentile at age 3 (i.e., underweight, average, overweight, obese) were tested as potential moderators of the model by stratifying analyses by each construct and using chi-square difference testing to compare the model with paths constrained within each group to freed paths. If chi-square difference testing indicates that allowing paths to freely vary between groups demonstrates significantly higher model fit, the model by grouping construct was be examined.

Full information maximum likelihood estimation was used to handle missing data [51]. Missing data information is presented in Supplementary Table 1. Indicator variables were centered [52]. Model fit was evaluated using three indices: root mean square error of approximation (RMSEA), comparative fit index (CFI), and a standardized root mean square residual (SRMR) [53]. Conventional cutoff criteria were applied, where good fit was indicated by RMSEA<.06, CFI > .95, SRMR<.08, and acceptable fit was indicated by RMSEA <.10, CFI > .90, and SRMR<.10 [54, 55]. Indirect effects are a statistical method to examine mediation [56]. We tested potential indirect effects between cumulative risk, sleep, and BMI from 3 to 9 years using a bootstrap estimation approach with 1000 samples and considered statistically significant if the 95% confidence interval did not contain zero [57].

## Results

Table 1 shows demographic variables and descriptive statistics for constructs of interest at each timepoint. Pearson’s correlation coefficients between main study variables of interest are presented in Table 3. Correlations between single risk indicators and child outcomes are presented in Supplementary Table 2.

**Fig. 1:**
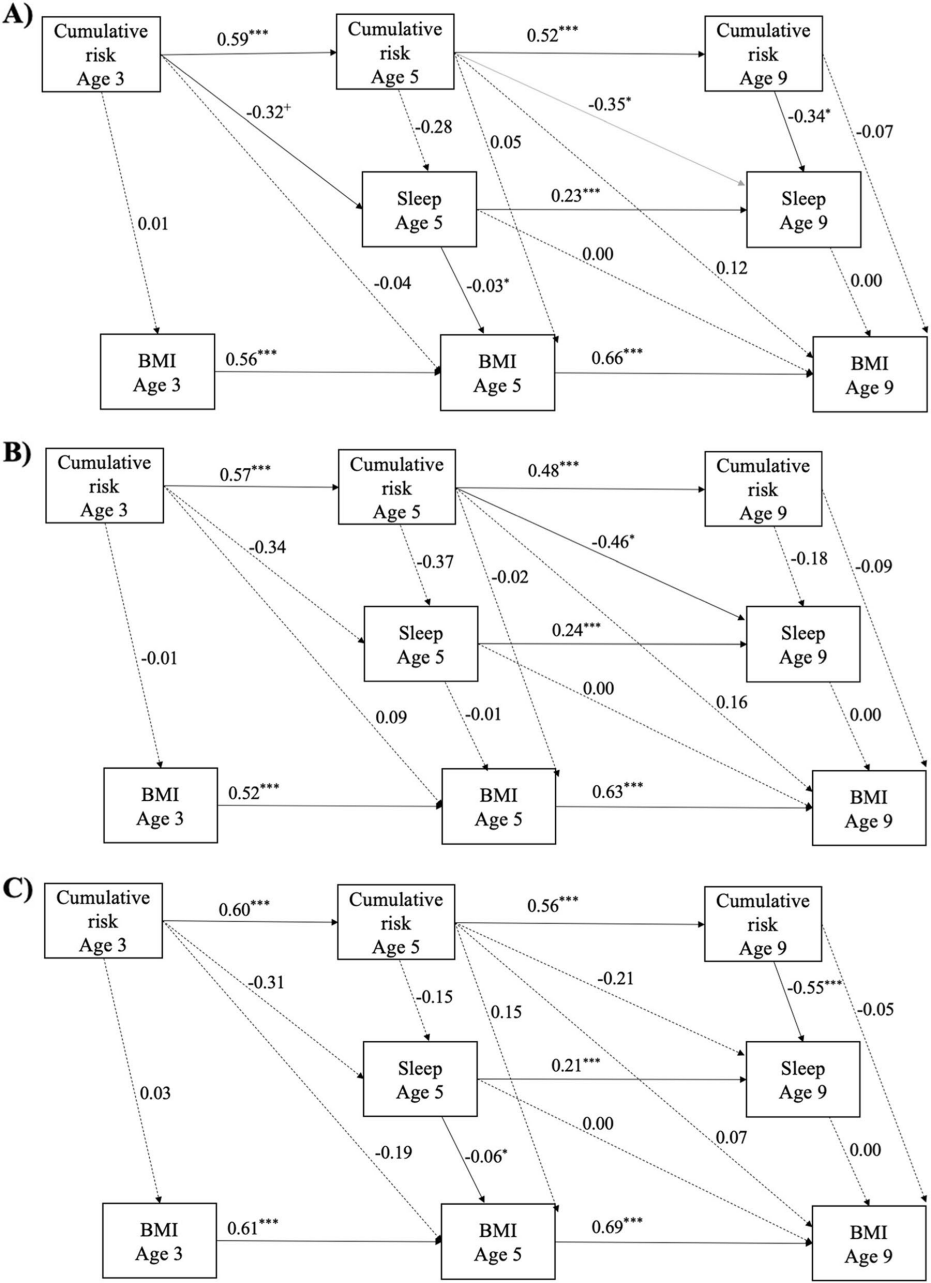
Structural equation model with autoregressive and lagged paths. Standardized coefficients are shown on each arrow; solid lines indicate statistically significant paths. Panel **A** presents the full model. Panel **B** presents the model for female children and Panel **C** for male children. Note: *** = *p* < .001, ** = *p* < .01, * = *p* < .05, + = *p* < .10; BMI = body mass index

The final structural equation model testing relationships between cumulative risk, sleep, and BMI demonstrated acceptable fit, RMSEA = 0.05 (90% CI [0.05, 0.06]), CFI = 0.91, SRMR = 0.04 (Fig. 1, Table 4). Dummy-coded maternal race/ethnicity (using Black participants as referent group), child low birthweight, child sex, and child age were entered as covariates on each construct at each timepoint. Autoregressive paths for BMI, cumulative risk, and sleep demonstrated positive relationships between measurements across timepoints, b’s = 0.23-0.67, *p*’s < .001. Two statistically significant concurrent paths emerged: longer child sleep duration at year 5 was concurrently associated with lower child BMI, b = − 0.03, *p* = .03, 95% CI [− 0.06, − 0.01], and higher cumulative risk at year 9 was concurrently associated with shorter child sleep duration at year 9, b = − 0.34, *p* = .02, 95% CI [− 0.57, − 0.10]. For lagged paths, higher cumulative risk at year 5 was associated with shorter child sleep duration at year 9, b = − 0.35, *p* = .01, 95% CI [− 0.57, − 0.11]. Lagged cumulative risk and sleep duration from the prior time point did not predict child BMI at the following wave. Indirect effects from age 3 to age 9 variables were not significant, *p’*s > .05. This model accounted for 33% of the variance in BMI at year 5 and 46% of the variance in BMI at year 9.

**Table 4:** Path analyses for the full sample and by child sex

	Full			Female			Male		
	b	95% CI	*p*-value	b	95% CI	*p-*value	b	95% CI	*p-*value
Autoregressive paths									
BMI y3 → BMI y5	0.56^***^	0.53, 0.60	<.001	0.52^***^	0.47, 0.57	<.001	0.61^***^	0.57, 0.66	<.001
BMI y5 → BMI y9	0.66^***^	0.63, 0.69	<.001	0.63^***^	0.60, 0.67	<.001	0.69^***^	0.65, 0.74	<.001
Risk y3 → Risk y5	0.59^***^	0.56, 0.61	<.001	0.57^***^	0.53, 0.60	<.001	0.60^***^	0.57, 0.64	<.001
Risk y5 → Risk y9	0.52^***^	0.50, 0.55	<.001	0.48^***^	0.45, 0.52	<.001	0.56^***^	0.52, 0.59	<.001
Sleep y5 → Sleep y9	0.23^***^	0.19, 0.26	<.001	0.24^***^	0.19, 0.28	<.001	0.21^***^	0.16, 0.25	<.001
Lagged paths									
Risk y3 → BMI y5	−0.04	−0.25, 0.15	.73	0.09	−0.19, 0.37	.60	−0.19	−0.47, 0.11	.27
Risk y5 → BMI y9	0.12	−0.04, 0.28	.21	0.16	−0.06, 0.37	.23	0.07	−0.17, 0.31	.62
Sleep y5 → BMI y9	0.00	−0.02, 0.02	.96	0.00	−0.03, 0.03	.93	0.00	−0.03, 0.03	.99
Risk y3 → Sleep y5	−0.32^+^	−0.60, −0.05	.05	−0.34	−0.74, 0.02	.13	−0.31	−0.72, 0.05	.19
Risk y5 → Sleep y9	− 0.35^*^	− 0.57, − 0.11	.01	− 0.46^*^	− 0.75, − 0.13	.02	− 0.21	− 0.55, 0.13	.32
Concurrent paths									
Risk y3 → BMI y3	0.01	− 0.18, 0.20	.94	− 0.01	− 0.26, 0.24	.94	0.03	− 0.20, 0.30	.83
Risk y5 → BMI y5	0.05	− 0.15, 0.26	.68	− 0.02	− 0.31, 0.27	.91	0.15	− 0.14, 0.45	.41
Risk y5 → Sleep y5	− 0.28	− 0.59, 0.01	.12	− 0.37	− 0.79, 0.02	.14	− 0.15	− 0.57, 0.29	.59
Sleep y5 → BMI y5	−0.03^*^	− 0.06, − 0.01	.03	−0.01	− 0.04, 0.02	.59	− 0.06^*^	−0.10, − 0.02	.02
Risk y9 → BMI y9	−0.07	− 0.24, 0.09	.48	− 0.09	−0.30, 0.12	.51	−0.05	− 0.31, 0.18	.73
Risk y9 → Sleep y9	− 0.34^*^	−0.57, − 0.10	.02	−0.18	− 0.52, 0.15	.37	− 0.55^*^	−0.91, − 0.20	.01
Sleep y9 → BMI y9	0.00	− 0.02, 0.02	.97	0.00	−0.03, 0.02	.85	0.00	−0.03, 0.04	.88

*** *p* < .001, ** *p* < .01, * *p* < .05, + *p* < .10. The “Full” column presents results of the path analysis covarying for dummy-coded maternal race/ethnicity (referent group = Black), child sex, child age, and low child birthweight on each construct at each timepoint. RMSEA = 0.05 [90% CI, 0.05, 0.06], CFI = 0.91, SRMR = 0.04. Path analyses stratified by child sex are presented under the “Female” and “Male” columns. This model allowed paths to freely vary between child sex and covaried for dummy-coded maternal race/ethnicity, child age, and low child birthweight. RMSEA = 0.05 [90% CI 0.04, 0.05], CFI = 0.93, SRMR = 0.04

Although there were statistically significant differences in child age, cumulative risk, and child BMI across maternal racial/ethnic groups (Supplementary Table 3), there were no differences in paths between cumulative risk, sleep duration, and BMI as a function of maternal race/ethnicity, Δχ^2^(27) = 8.37, *p* = .99. In addition, the final model did not differ by categorical child BMI status at year 3 (underweight, average, overweight/obese), Δχ^2^(82) = 92.35, *p = .*20. However, the model did differ by child sex, Δχ^2^(36) = 65.70, *p* = .002 (see Fig. 1). Model fit was adequate, RMSEA = 0.05 (90% CI [0.04, 0.05]), CFI = 0.93, SRMR = 0.04. For female children, higher cumulative risk at year 5 was associated with shorter sleep duration at year 9, b = − 0.46, *p* = .02. For male children, longer sleep duration at year 5 was concurrently associated with lower BMI, b = − 0.06, 95% CI [− 0.10, − 0.02], *p* = .02, and higher cumulative risk at age 9 was concurrently associated with lower sleep duration, b = − 0.55, 95% CI [− 0.91, − 0.20], *p* = .01.

## Discussion

This study examined transactional and cascading associations between cumulative risk, sleep duration, and BMI across childhood within a national sample of families experiencing relatively high socioeconomic risk who were from a range of racial/ethnic backgrounds. The prevalence of obesity in the FFCWS sample appeared slightly higher compared to national estimates in 2017-2018 [58], where 18% of children at age 3 were categorized as obese in this sample compared to national estimates of 13% for those ages 2-4 and 26% at age 9 in this sample compared to national estimates of 20% for those ages 5-11. Novel contributions include modeling cumulative risk across several indices at individual timepoints to capture fluctuations in risk across childhood. Prior research suggests that families cycle in and out of childhood poverty [59], which can precede or accompany many facets of cumulative risk (e.g., household insecurity, food insecurity, substandard housing). Therefore, examining the direction of associations between cumulative risk, sleep health and physical health requires dynamically capturing these stressors across childhood. Additionally, the lagged, autoregressive paths in our statistical model allows us to account for effects of measured constructs at earlier timepoints and investigate what accounts for variability in each construct across time.

Findings differed by child sex. Although both female and male children showed relationships between higher cumulative risk and shorter sleep duration, this finding emerged for female children as a lagged relationship (from age 5 to 9) and male children as a concurrent relationship (at age 9). Responsivity to stressors may mature with different developmental timing by sex [60]. Only male children in the current study showed a concurrent relationship between longer sleep duration and lower BMI, which occurred at age 5. This finding aligns with another study of children in middle childhood, which found that only male children showed a relationship between sleep duration and obesity incidence [61]. It is unclear why this gender-specific effect was replicated within a younger age; potential factors that warrant further study include sex hormones and feeding and eating behaviors that may be socialized differentially based on sex [62].

Associations emerging between cumulative risk and sleep duration align with another study examining longitudinal sleep quality across childhood, which found that the presence of sociodemographic risk in early childhood is related to poorer sleep health, pediatric insomnia symptoms, and obstructive sleep apnea symptoms [14] and increased sleep problems in middle childhood [63]. Several components captured in the cumulative risk composite used here include environmental characteristics that negatively affect sleep health and sleep-related behaviors. For example, noisiness is associated with poorer attained sleep quality [64], substandard housing is associated with increased sleep disturbances [65], and housing insecurity is associated with less consistent bedtime routines [66]. Beyond physical characteristics, increased parental stress associated with high cumulative risk may strain parents’ abilities to facilitate child sleep health [61].

Longer sleep duration at age 5 demonstrated a small, concurrent relationship with lower BMI for male children, suggesting that parents’ ability to promote sleep health in early childhood and lower weight are related for this group (although the directionality is unclear). One study examining effects of a 6-month preschool weight management intervention found that increasing sleep duration yielded lower BMI, lower caloric intake, and reduced consumption of added sugars [67]. This intervention conducted psychoeducation on structured bedtime routines and sleep hygiene, with subsequent goal-setting and support in implementing changes [68]. In context of the current study’s finding that higher cumulative risk was related to shorter sleep duration, it is possible that sleep-related behaviors mediate this association.

This study investigated patterns of cumulative risk, sleep duration, and BMI from ages 3 to 9 using three timepoints within a large, national dataset oversampled for single mothers. No statistically significant indirect effects were found in the full sample, indicating lack of evidence supporting our initial hypothesis of cascading effects between cumulative risk, sleep duration, and BMI in the FFCWS sample across ages 3 to 9. When examining models stratified by child sex, only male children showed that shorter sleep duration was related to higher BMI, occurring at age 5. For female children, higher cumulative risk at year 5 was associated with shorter sleep duration at year 9. Contrary to Suglia and colleagues [28] who found associations in early childhood between cumulative social risk and increased odds of obesity at year 5 among female children, cumulative risk was not directly related to BMI in our analyses. We speculate that using path analyses in the current study to account for correlations between cumulative risk and sleep may have decreased the pool of variance attributable to BMI.

The model did not significantly differ by maternal race/ethnicity, suggesting that relations between cumulative risk, sleep duration, and BMI in childhood are similar across racial/ethnic groups. This is consistent with other work indicating that race/ethnicity did not moderate associations between adverse childhood experiences and insulin resistance among adolescents [69]. As examined pathways did not differ by racial/ethnic group in the current sample, differential exposure to stressors and adversity may have driven group-level differences in weight status. Within the current study, Black and Latinx groups showed higher cumulative risk exposure across timepoints compared to White and Other groups. Future directions include examining factors that underpin this differential exposure to cumulative risk by racial/ethnic groups and additionally capturing experiences of parental perceived discrimination as an important and relevant stressor potentially impacting child physical health and sleep. Supporting the importance of examining racism and discrimination in relation to child health, one large epidemiological study indicates that increased maternal experiences of racism were associated with higher child BMI and poorer socioemotional outcomes [70].

Strengths of the current study include repeated measurement of each construct over time to capture constructs as they change over time and potentially affect one another (e.g., maternal education, housing quality, exposure to violence, presence of cohabitating or stable romantic partner). Our investigation of cumulative risk, sleep, and BMI builds on prior work within this sample by broadly encapsulating risk across relevant contexts, as supported by Bronfenbrenner’s bioecological theory [3]. Using multiple timepoints for each construct and modeling both autoregressive and lagged paths allows our work to distinguish trajectories over time from how changes in constructs are related to each other. When utilizing this broader measure of cumulative risk in our model and examining BMI using z-scored percentile, we replicated Suglia et al. [28]’s finding of a concurrent association between shorter sleep duration and increased likelihood of experiencing obesity at age 5. Suglia and colleagues [28] noted that only female children showed relationships between higher cumulative social risk at ages 1 or 3 and increased odds of experiencing obesity at age 5, while whereas we only found concurrent relationships between sleep duration and BMI for male children.

Limitations of the current study include only using one indicator of sleep health (parent-reported child sleep duration) and lack of sleep duration data at the age 3 timepoint, which restricts fully capturing relationships between sleep health, cumulative risk, and BMI over time. For example, other dimensions of sleep health may have stronger biological underpinnings of endocrine and metabolic systems related to hunger, satiety, and weight, which could be affected by cumulative risk and sleep. One systematic review of sleep health dimensions and weight in childhood found that sleep timing and sleep efficiency may importantly influence pediatric obesity [71]. Future directions should capture other components of sleep using tools such as actigraphy, polysomnography, obstructed breathing patterns, and standardized child sleep questionnaires.

The current study is limited in its focus on BMI as the sole indicator of child physical health. Although BMI is a well-studied construct with demonstrated links to later cardiovascular health [72], utilizing multiple indicators of physical health will be important in future research. Research examining effects of cumulative risk and sleep health on physical health should incorporate multiple measures of physical health, such as blood pressure, waist circumference, and serum markers of cardiovascular risk such as insulin resistance and triglyceride levels.

In this sample, higher cumulative risk showed some prospective and concurrent associations with shorter sleep duration. Shorter sleep duration also showed an independent association with higher BMI at age 5 for male children, but not female children. Implications of these findings include the potential for pediatric sleep health interventions to positively shape child weight status, particularly for male children, and suggest that the presence of increased cumulative risk may impair healthy sleep practices. Several variables that comprised the cumulative risk composite used here are directly shaped by federal and state policies, such as housing insecurity, food insecurity, substandard housing, high school equivalency programs, and income-to-needs ratio. Some evidence suggests that supporting employment and income reduces poverty-related cumulative risk (e.g., material hardship, depressive symptoms, marital status, parenting stress) [73]. A naturalistic study in Alaska demonstrated that providing families with universal income was associated with decreased child BMI in early childhood [74]. Future work reducing cumulative risk in families should measure child sleep and BMI to understand whether improving the environment improves these measures of child health as a result. Although capturing high levels of risk exposure across relevant domains is a strength of the cumulative risk approach, studies examining individual risk factors will complement studies on cumulative risk and elucidate particularly important areas for intervention.

## Supplementary Information

**Additional file 1: Supplementary Table 1.** Percentage of missing data for the full analytic sample. **Supplementary Table 2.** Correlations between single risk indicators and child parameters of interest. **Supplementary Table 3.** Descriptives for child age, cumulative risk, BMI percentile, and sleep duration between racial/ethnic groups, along with ANOVA tests for group differences. **Supplementary Table 4.** Descriptives for child age, cumulative risk, BMI percentile and sleep duration by child sex, along with t-tests for group differences.

## Data Availability

The data analyzed during the current study are publicly available for download from Princeton University’s Office of Population Research data archive [https://opr.princeton.edu/archive/restricted/Default.aspx].

## Abbreviations

BMI: Body Mass Index
CDC: Center for Disease Control and Prevention
CFI: Comparative Fit Index
CI: Confidence Interval
CIDI: Composite International Diagnostic Interview- Short Form
FFCWS: Fragile Families and Child Wellbeing Study
RMSEA: Root Mean Square Error of Approximation
SRMR: Standardized Root Mean Residual
