# Supplementary material for: Associations between cumulative risk, childhood sleep duration, and body mass index across childhood
Source: BMC Pediatr. 2022 Sep 6;22:529. doi: 10.1186/s12887-022-03587-6 (PMC9447344; doi:10.1186/s12887-022-03587-6)
Supplement: Supplementary file 1 — Additional file 1: Supplementary Table 1. Percentage of missing data for the full analytic sample. Supplementary Table 2. Correlations between single risk indicators and child parameters of interest. Supplementary Table 3. Descriptives for child age, cumulative risk, BMI percentile, and sleep duration between racial/ethnic groups, along with ANOVA tests for group differences. Supplementary Table 4. Descriptives for child age, cumulative risk, BMI percentile and sleep duration by child sex, along with t-tests for group differences. [file 12887_2022_3587_MOESM1_ESM.docx]

Supplementary Appendix

**Supplementary Table 1.**

Percentage of missing data for the full analytic sample.

|  | Age 3 (n=3451) | Age 5 (n=3419) | Age 9 (n=3229) |
| --- | --- | --- | --- |
| BMI | 32.3% (n=1115)^a^ | 38.6% (n=1319)^b^ | 2.8% (n=91) |
| Sleep duration | - | 21.2% (n=724)^c^ | 0.7% (n=22) |
| Housing conditions | 44.0% (n=1519) | 40.9% (n=1397) | 4.3% (n=140) |
| Total household members | 6.9% (n=255) | 0.6% (n=19) | 0.5% (n=16) |
| Noisy | 51.1% (n=1765) | 41.8% (n=1428) | 5.2% (n=167) |
| Child separation | 4.2 (n=144) | 5.1% (n=176) | 6.0% (n=195) |
| Child exposure to violence | 26.1% (n=901) | 20.2% (n=689) | 12.2% (n=393) |
| Family turmoil | 0.0% (n=0) | 0.0% (n=0) | 0.0% (n=0) |
| Maternal education | 0.1% (n=2) | 0.1% (n=2) | 0.0% (n=1) |
| Single mother | 0.1% (n=5) | 0.1% (n=2) | 1.1% (n=36) |
| Income-to-needs ratio | 0.0% (n=0) | 0.0% (n=0) | 0.0% (n=0) |
| Low birthweight | 0.0% (n=0) | 0.0% (n=0) | 0.0% (n=0) |
| Maternal race/ethnicity | 0.0% (n=0) | 0.0% (n=0) | 0.0% (n=0) |
| Child sex | 0.0% (n=0) | 0.0% (n=0) | 0.0% (n=0) |

Notes:

1. Comparing those with missing versus present BMI data at year 3 across the FFCWS sample indicated no differences in child sex, χ^2^(1)=.80, p=.37. Participants with and without BMI at year 3 did differ on income-to-needs ratio, t(4857)=2.71, p=.01 (with year 3 BMI data, M=2.13, SD=2.37; without, M=2.32, SD=2.46), maternal education, χ^2^(1)=3.26, p=.07 (with year 3 BMI data, 64% high school and above; without, 67%), and maternal race/ethnicity, χ^2^(3)=41.73, p<.001 (with year 3 BMI data: 52% non-Hispanic Black, 25% Latina, 20% non-Hispanic White, 3% Other; without year 3 BMI data: 43% non-Hispanic Black, 29% Latina, 23% non-Hispanic White, 5% Other).
2. Comparing those with missing versus present BMI data at year 5 across the FFCWS sample indicated no differences in maternal education, χ^2^(1)=0.04, p=.95. Participants with and without BMI at year 5 did differ in child sex, χ^2^(1)=.3.14, p=.08 (with year 5 BMI data: 51% male; without, 53% male), income-to-needs ratio, t(4737)=2.63, p=.01 (with year 5 BMI data, M=2.12, SD=2.30; without, M=2.30, SD=2.49), and maternal race/ethnicity, χ^2^(3)=47.46, p<.001 (with year 5 BMI data: 52% non-Hispanic Black, 25% Latina, 20% non-Hispanic White, 3% Other; without year 5 BMI data: 43% non-Hispanic Black, 30% Latina, 22% non-Hispanic White, 5% Other).
3. Comparing those with missing versus present sleep data at year 5 across the FFCWS sample indicated no differences in income-to-needs ratio, t(4895)=1.28, p=.20, and child sex, χ^2^(1)=.61, p=.44. Participants with and without sleep data at year 5 did differ on maternal education, χ^2^(1)=4.59, p=.03 (with year 5 sleep data, 66% high school and above; without, 64%) maternal race/ethnicity, χ^2^(3)=58.52, p<.001 (with year 5 sleep data: 51% non-Hispanic Black, 25% Latina, 21% non-Hispanic White, 3% Other; without year 5 sleep data: 42% non-Hispanic Black, 31% Latina, 21% non-Hispanic White, 6% Other).

**Supplementary Table 2.**

Correlations between single risk indicators and child parameters of interest

| Risk indicators | BMI y3 | BMI y5 | BMI y9 | Sleep duration y5 | Sleep duration y9 |
| --- | --- | --- | --- | --- | --- |
| Substandard housing y3 | .02 | .04 | .04 | -.02 | .05^*^ |
| Substandard housing y5 | -.01 | .02 | .03 | -.11^***^ | -.07^**^ |
| Substandard housing y9 | .02 | .03 | .00 | .01 | -.04^*^ |
| Household size y3 | .03 | .03 | .02 | .00 | .01 |
| Household size y5 | .02 | -.01 | -.02 | .03^*^ | .01 |
| Household size y9 | -.02 | -.05^*^ | -.06^***^ | .02 | .10^***^ |
| Noisy y3 | .01 | .00 | .00 | .00 | -.06^*^ |
| Noisy y5 | .02 | .02 | .01 | -.08^***^ | -.04 |
| Noisy y9 | -.05^*^ | .00 | .02 | -.02 | -.07^***^ |
| Child separation y3 | -.04 | .01 | .02 | -.04^**^ | -.12^***^ |
| Child separation y5 | -.01 | -.03 | .01 | -.06^***^ | -.10^***^ |
| Child separation y9 | -.03 | .00 | .01 | -.09^***^ | -.10^***^ |
| Violence exposure y3 | -.01 | -.01 | -.02 | .00 | -.01 |
| Violence exposure y5 | .00 | -.02 | -.01 | .02 | .03 |
| Violence exposure y9 | .04 | .03 | .02 | -.03 | -.09^***^ |
| Family turmoil y3 | -.02 | .01 | -.02 | .03^+^ | .04^*^ |
| Family turmoil y5 | -.03 | -.01 | -.01 | .05^**^ | .04^*^ |
| Family turmoil y9 | .02 | .04^+^ | .04^*^ | .02 | .06^**^ |
| Maternal education less than high school y3 | .04^+^ | .01 | .03 | -.03 | -.06^***^ |
| Maternal education less than high school y5 | .04^+^ | .02 | .02 | .00 | -.07^***^ |
| Maternal education less than high school y9 | .04^+^ | -.01 | .02 | -.02 | -.07^***^ |
| Single mother y3 | .01 | .03 | .05^**^ | .04^*^ | .04^*^ |
| Single mother y5 | .00 | .05^*^ | .06^***^ | .03^+^ | .02 |
| Single mother y9 | .00 | .06^**^ | .03^+^ | -.02 | -.10^***^ |
| Income to needs ratio y3 | .00 | -.02 | -.06^***^ | -.03^+^ | .02 |
| Income to needs ratio y5 | .04^+^ | .00 | -.05^**^ | -.03^*^ | .04^*^ |
| Income to needs ratio y9 | .00 | -.03 | -.05^**^ | .00 | .11^***^ |

Notes: y3= child aged 3 years (Wave 3), y5= child aged 5 years (Wave 4), y9= child aged 9 years (Wave 5). * p<.05, ** p<.01, *** p<.001. With exception of income to needs ratio, all indicators have been coded such that higher scores indicate greater risk.

**Supplementary Table 3.**

Descriptives for child age, cumulative risk, BMI percentile, and sleep duration between racial/ethnic groups, along with ANOVA tests for group differences.

|  |  | Racial/ethnic groups  M (SD) | | | | Group differences |
| --- | --- | --- | --- | --- | --- | --- |
| Construct |  | White | Black | Latinx | Other |  |
| Child age (years) | Year 3 | 2.92 (0.19) | 3.00 (0.21) | 3.00 (0.23) | 2.98 (0.22) | F(3, 4216)=35.68, p<.001 |
|  | Year 5 | 5.10 (0.22) | 5.16 (0.22) | 5.19 (0.26) | 5.16 (0.26) | F (3, 4126)=27.33, p<.001 |
|  | Year 9 | 9.33 (0.33) | 9.37 (0.38) | 9.47 (0.39) | 9.45 (0.46) | F (3, 3504)=22.52, p<.001 |
| Cumulative risk | Year 3 | 0.14 (0.17) | 0.25 (0.19) | 0.25 (0.19) | 0.17 (0.19) | F(3, 4214)=107.71, p<.001 |
|  | Year 5 | 0.12 (0.17) | 0.24 (0.18) | 0.23 (0.18) | 0.14 (0.17) | F (3, 4125)=103.26, p<.001 |
|  | Year 9 | 0.12 (0.15) | 0.23 (0.17) | 0.22 (0.17) | 0.14 (0.15) | F (3, 3561)=83.96, p<.001 |
| BMI percentile | Year 3 | 62.68 (29.62) | 60.69 (30.86) | 70.18 (29.90) | 60.70 (31.72) | F(3, 2406)=13.68, p<.001 |
|  | Year 5 | 63.99 (29.03) | 64.60 (28.97) | 71.56 (27.02) | 61.18 (28.53) | F (3, 2155)=8.89, p<.001 |
|  | Year 9 | 62.81 (29.86) | 70.79 (27.72) | 73.09 (27.56) | 66.11 (28.73) | F (3, 3338)=19.34, p<.001 |
| Sleep duration | Year 5 | 9.85 (1.17) | 9.15 (1.27) | 9.56 (1.24) | 9.75 (0.97) | F (3, 2963) = 54.28, p<.001 |
|  | Year 9 | 9.34 (1.02) | 8.74 (1.13) | 9.07 (1.04) | 8.95 (0.97) | F (3, 3612) = 61.54, p<.001 |

**Supplementary Table 4.**

Descriptives for child age, cumulative risk, BMI percentile and sleep duration by child sex, along with t-tests for group differences.

|  |  | Child sex | | Group Differences |
| --- | --- | --- | --- | --- |
| Construct |  | Male | Female |  |
| Child age (years) | Year 3 | 2.98 (0.21) | 2.98 (2.22) | t (4229) = 0.02, p=.98 |
|  | Year 5 | 5.15 (0.23) | 5.16 (0.24) | t (4137)= -1.41, p=.16 |
|  | Year 9 | 9.38 (0.38) | 0.40 (0.38) | t (3513) = -0.96, p=.34 |
| Cumulative risk | Year 3 | 0.22 (.20) | 0.23 (0.19) | t (4227)= -0.84, p=.40 |
|  | Year 5 | 0.21 (0.19) | 0.21 (0.18) | t (4136)= 0.85, p=.40 |
|  | Year 9 | 0.20 (0.17) | 0.20 (0.17) | t (3570)= -1.23, p=.22 |
| BMI percentile | Year 3 | 63.28 (30.54) | 63.64 (30.80) | t (2414)= -0.29), p=.77 |
|  | Year 5 | 65.77 (28.52) | 66.43 (28.78) | t (2161)= -0.54, p=.59 |
|  | Year 9 | 69.62 (27.61) | 69.49 (29.27) | t (3347)=0.13, p=.90 |
| Sleep duration | Year 5 | 9.42 (1.27) | 9.42 (1.27) | t (2971)= -0.06, p=.96 |
|  | Year 9 | 8.94 (1.10) | 8.97 (1.12) | t (3621)= - 0.86, p=.39 |
